# Theoretical Study of the Structures of 4-(2,3,5,6-Tetrafluoropyridyl)Diphenylphosphine Oxide and Tris(Pentafluorophenyl)Phosphine Oxide: Why Does the Crystal Structure of (Tetrafluoropyridyl)Diphenylphosphine Oxide Have Two Different P=O Bond Lengths?

**DOI:** 10.3390/molecules25122778

**Published:** 2020-06-16

**Authors:** Joseph R. Lane, Graham C. Saunders

**Affiliations:** School of Science, University of Waikato, Hamilton 3240, New Zealand; joseph.lane@waikato.ac.nz

**Keywords:** phosphine oxides, X-ray crystal structure, density functional theory calculations

## Abstract

The crystal structure of 4-(2,3,5,6-tetrafluoropyridyl)diphenylphosphine oxide (**1**) contains two independent molecules in the asymmetric unit. Although the molecules are virtually identical in all other aspects, the P=O bond distances differ by ca. 0.02 Å. In contrast, although tris(pentafluorophenyl)phosphine oxide (**2**) has a similar crystal structure, the P=O bond distances of the two independent molecules are identical. To investigate the reason for the difference, a density functional theory study was undertaken. Both structures comprise chains of molecules. The attraction between molecules of **1**, which comprises lone pair–π, weak hydrogen bonding and C–H∙∙∙arene interactions, has energies of 70 and 71 kJ mol^−1^. The attraction between molecules of **2** comprises two lone pair–π interactions, and has energies of 99 and 100 kJ mol^−1^. There is weak hydrogen bonding between molecules of adjacent chains involving the oxygen atom of **1**. For one molecule, this interaction is with a symmetry independent molecule, whereas for the other, it also occurs with a symmetry related molecule. This provides a reason for the difference in P=O distance. This interaction is not possible for **2**, and so there is no difference between the P=O distances of **2**.

## 1. Introduction

**Figure 1:**
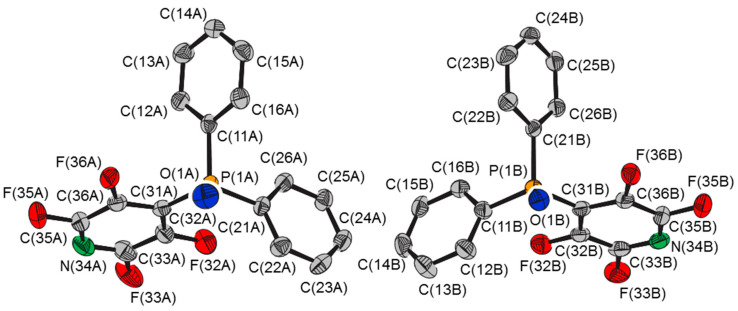
Molecular structures of the two independent molecules of 4-(2,3,5,6-tetrafluoropyridyl)diphenylphosphine oxide, **1**.

The crystal structure of 4-(2,3,5,6-tetrafluoropyridyl)diphenylphosphine oxide, **1** (CSD ref. code: TACWIE) [1] crystallized in the space group *P*2_1_/c with two symmetry independent molecules in the asymmetric unit (Zʹ = 2, *R*_1_ = 0.0517). The two independent molecules possess virtually identical conformations (Figure 1), with very similar geometric parameters (Table 1), but the P=O bond lengths differ by ca. 0.02 Å. Clearly, this is not an example of bond-stretch isomerism, whereby molecules differ only in the length of one or more bonds [2]; the structures of the two molecules, although virtually identical, do exhibit slight differences in some of the bond and torsion angles (Table 1). Nor is it likely to be a case of an apparent change of bond length caused by the presence of an isomorphous impurity [3]; the major axes of the displacement ellipsoids of the oxygen atoms of **1** are perpendicular to the P=O bond, not co-linear, the common valencies of phosphorus do not support a P–X bond in place of the P=O bond, and the manner of formation, aerial oxidation, was very unlikely to have generated anything other than the oxide [1]. It is not unexpected that symmetry independent molecules of the same compound within a crystal structure possess different structures [4], but typically, bond lengths are identical within experimental error. About 280 good quality crystal structures of phosphine oxides in the Cambridge Structural Database [5] with more than one molecule in the asymmetric unit fewer than 0.5% possess significantly different P=O distances. The majority of those with different P=O distances display intermolecular [6,7,8,9,10,11] or both intramolecular and intermolecular [12,13] P=O∙∙∙H–O hydrogen bonding. It is not unexpected that such a relatively strong interaction can affect the P=O bond length, and that slight variations in the environment of the interaction may lead to significant differences between symmetry independent molecules, as, for example, in the *C*c polymorph of triphenylphosphine hemihydrate (1.487(1) and 1.499(2) Å) (CSD ref. code: JEDTOB02) [10], and the co-crystal of triphenylphosphine oxide and triphenylsilanol (1.493(1) and 1.503(1) Å) (CSD ref. code: ZOQHIY) [11]. In the crystal structure of di-*t*-butylphosphorylmethyl-4-methylbenzenesulfonate (CSD ref. code: KIMCUG), the different environments of the oxygen atoms provide a reason for a difference in P=O bond length, 1.491(1) cf. 1.499(1) Å: the molecules with the shorter P=O bonds (1.491(1) Å) form dimers with the shortest O∙∙∙C(tolyl) distance 3.239(2) Å, whereas the molecules with the longer P=O bonds (1.499(1) Å) are arranged head-to-tail in chains with short O∙∙∙C(butyl) distances of 3.400(2), 3.737(2) and 3.841(2) Å [14]. In addition to that of **1**, only two other crystal structures possess oxygen atoms in similar environments and no P=O∙∙∙H–O hydrogen bonding. Both are organometallic complexes that show other significant differences between the independent molecules: the dinuclear cobalt complex [(μ–P,P–PPh_2_CH_2_PPh_2_)Co_2_(CO)_4_{μ,η-Me_2_NCH_2_C≡CPO*t*Bu_2_}] (P=O 1.449(3) and 1.490(2) Å) (QOHPOT) [15], for which some other bond distances and angles differ considerably (e.g., Co-Co 2.4562(6) and 2.4782(6) Å, Co–P 2.2339(9) and 2.2428(8), and 2.2360(8) and 2.234(9) Å, OC–Co–CO 100.7(2) and 103.2(2), and 102.1(2) and 96.1(2)°), and the ferrocenophane [Fe{(η–C_5_H_4_)_2_PO(CH_2_SiMe_3_)}] (P=O 1.427(2) and 1.479(2) Å) (GUTWIC) [16], for which the Cp*–Fe–Cp* angles differ by ca. 5° and the Fe–C-P=O torsion angles differ by ca. 3°.

**Figure 2:**
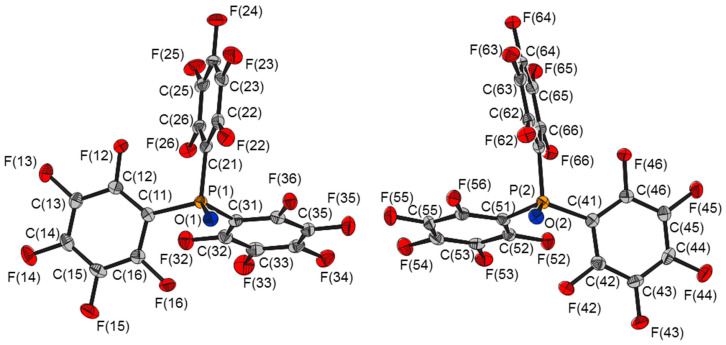
Molecular structures of the two independent molecules of tris(pentafluorophenyl)phosphine oxide, **2**.

Pentafluorophenyl(diphenyl)phosphine oxide (CSD ref. code: DPFPPO) [17]) possesses a structure similar to that of **1**, crystallizing in the *P*2_1_/c space group with Zʹ = 2, but although the P=O bond lengths were reported to be different, 1.469 and 1.479 Å, the data are of insufficient quality to determine whether this difference is genuine or not. The uncertainties for these distances were not given, but based on those of the unit cell lengths, which are four times greater than those for **1**, the reported P=O bond lengths are identical within experimental error. The related compound, tris(pentafluorophenyl)phosphine oxide (CSD ref. code: IMINUN) [18], **2** (Figure 2), also possesses a crystal structure similar to that **1**, crystallizing in the *P*2_1_/c space group with Zʹ = 2 (*R*_1_ = 0.0399), but with identical P=O bond lengths, 1.467(2) Å, and almost identical symmetry independent molecules (Table 2).

In order to investigate why the P=O bond distances of **1** are so different, whilst those of **2** are identical, we have undertaken density functional theory (DFT) calculations of the interactions between the molecules. Here, we report the results of our study.

## 2. Results and Discussion

**Figure 3:**
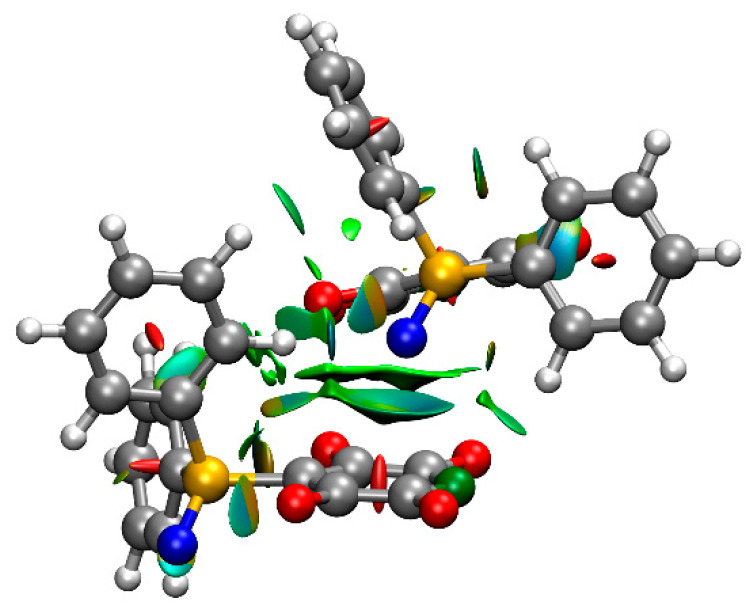
The NCI isosurfaces of the two independent molecules of **1** with *s* = 0.5 and a blue–green–red colour scale, to indicate the strength of the interaction from −0.02 a.u. (blue, strongly attractive) < sign(λ_2_) < +0.02 a.u. (red, strongly repulsive).

The structures of isolated molecules of **1** and **2** in the gas phase were optimized by the long-range corrected functional ωB97xD [19], using the 6-311G++(2d,2p) basis set. The bond distances and angles show reasonable agreement with the experimental values determined in the solid state (Table 1 and Table 2), with the exception of the P=O bond of **1**, for which the calculated distance is significantly longer. The experimental and calculated structures of **1** contain two short O∙∙∙C distances. The attractive nature of each of these interactions is indicated by the reduced density gradient (*s*) isosurfaces, calculated using non-covalent interactions (NCI) theory (Figure 3). Each isosurface volume corresponds to a unique non-covalent interaction, with the color scale denoting the strength of interaction. Those isosurfaces that are green denote weak attractive interactions, whereas the red isosurface at the center of the aryl rings denote the repulsive steric interaction due to ring formation. The plot also indicates a C–H∙∙∙arene interaction [20,21] between the two phenyl substituents, and attractive interactions between the ortho fluorine atoms and the phenyl rings.

**Figure 4:**
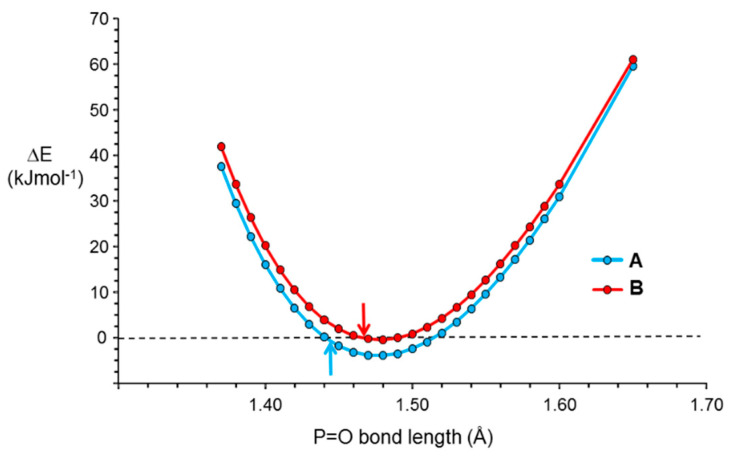
Plot of the variation of energy, relative to that of the experimentally determined structures, with the length of the P=O bond of the two independent molecules of 4-(2,3,5,6-tetrafluoropyridyl)diphenylphosphine oxide **1**. The structures of the molecules are fixed with the exception of the P=O bond lengths. The arrows indicate the energies of the experimental bond lengths.

The experimental structures of molecules **1A** and **1B** are respectively 30 and 28 kJ mol^−1^ higher in energy than the optimized structures. Optimization of the position of the oxygen atom for the experimental structures gives a P=O bond length of 1.468 Å for both structures and energies that are lower by 1 and 6 kJ mol^−1^ than those of **1A** and **1B** respectively. Variation of the P=O bond length between 1.44 and 1.52 Å, whilst keeping the other geometric parameters constant, gives only small changes in energy, < 5 kJ mol^−1^, with, as expected, a sharp increase, as P=O is shortened further and a slightly gentler increase, as P=O is lengthened above 1.52 Å (Figure 4).

**Figure 5:**
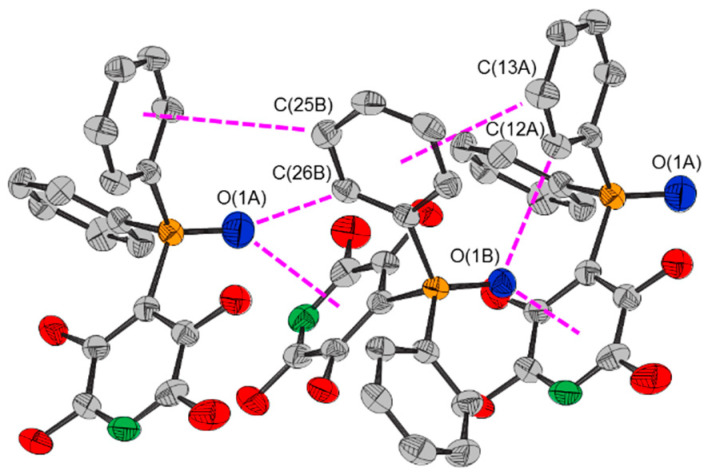
Intermolecular interactions between molecules of 4-(2,3,5,6-Table 1. within a chain.

The crystal structure of **1** possesses short contacts between adjacent non-identical molecules of opposite conformation (Table 3). In particular, the oxygen atom of one molecule is directed towards, and close to, one face of the tetrafluoropyridyl ring of an adjacent non-equivalent molecule, suggestive of a lone pair–π interaction [22,23,24] (Figure 5). It is also close to an ortho phenyl carbon atom, suggestive of weak intermolecular hydrogen bonding [25,26,27]. The adjacent meta carbon atom is close to the face of a phenyl ring of the other molecule, suggestive of a C–H∙∙∙arene interaction [20,21]. The attractive nature of each of these interactions is indicated by the reduced density gradient (*s*) isosurfaces calculated using NCI theory (Figure 3). The plot also indicates a weak attractive intermolecular interaction between an ortho hydrogen atom and the nitrogen atom of the tetrafluoropyridyl ring. The interactions between the adjacent molecules, as dimers in the gas phase, were calculated to be −70 and −71 kJ mol^−1^ for **1A** and **1B** respectively, possessing the interacting oxygen atom. As a consequence of these interactions the molecules are arranged in columns parallel to the *b* axis, with which the P=O bonds are virtually parallel (deviating by 4.0(1) and 3.6(1)° for molecules **A** and **B** respectively).

**Figure 6:**
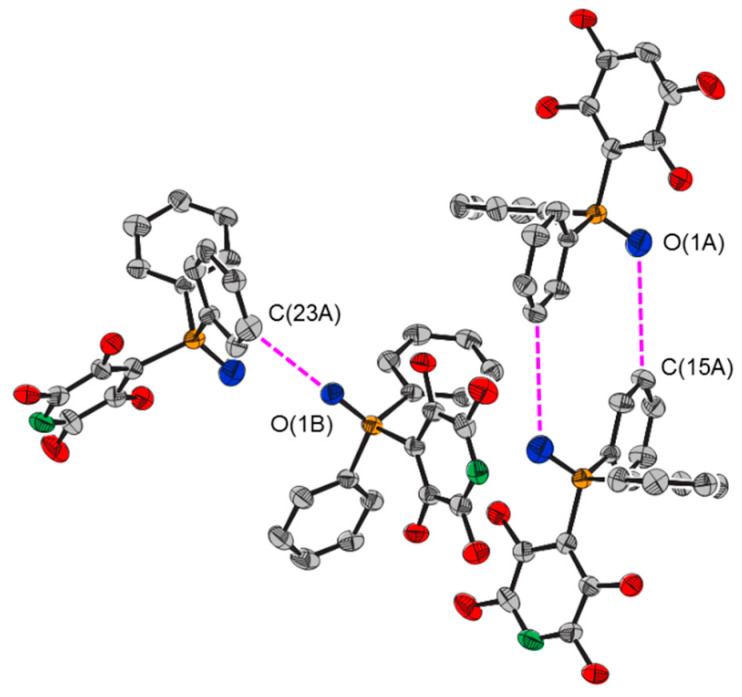
Intermolecular interactions between molecules of 4-(2,3,5,6-tetrafluoropyridyl)diphenylphosphine oxide, **1**, of different chains.

**Figure 7:**
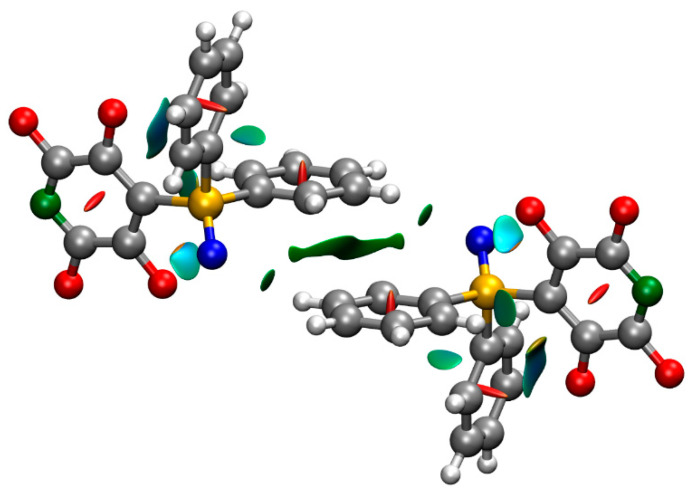
The NCI isosurface of a dimer two molecules of **1A** in different chains with *s* = 0.5 and a blue–green–red color scale to indicate the strength of the interaction from −0.02 a.u. (blue, strongly attractive) < sign(λ2) < +0.02 a.u. (red, strongly repulsive).

**Figure 8:**
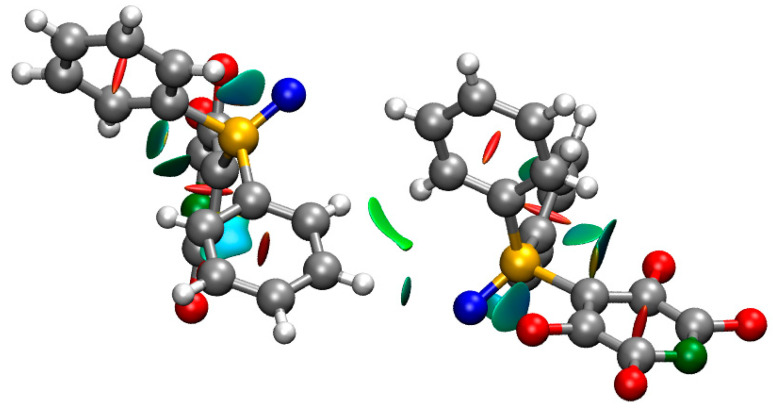
The NCI isosurface of a dimer of molecules **1A** and **1B** in different chains with *s* = 0.5 and a blue–green–red color scale to indicate the strength of the interaction from −0.02 a.u. (blue, strongly attractive) < sign(λ2) < +0.02 a.u. (red, strongly repulsive).

There is a short distance between the oxygen atom of one molecule and the meta hydrogen atom of a molecule of an adjacent chain (Figure 6), suggestive of hydrogen bonding between the molecules [25,26,27]. For **1A**, the contact is with another molecule of **1A**, with the two molecules related by a centre of inversion, such that there are two short O∙∙∙C(15A) distances. The interaction between the two molecules was calculated to be attractive by 33 kJ mol^−1^. The NCI isosurfaces indicated attractive C–H∙∙∙O interactions and an attractive interaction between the two parallel phenyl rings (Figure 7). For **1B**, there is just one contact, with a molecule of **1A** in a third chain (Figure 6). The interaction between the molecules was calculated to be attractive by 23 kJ mol^−1^, with the NCI isosurfaces indicating an attractive C–H∙∙∙O interaction and an attractive C–H∙∙∙arene interaction (Figure 8).

**Figure 9:**
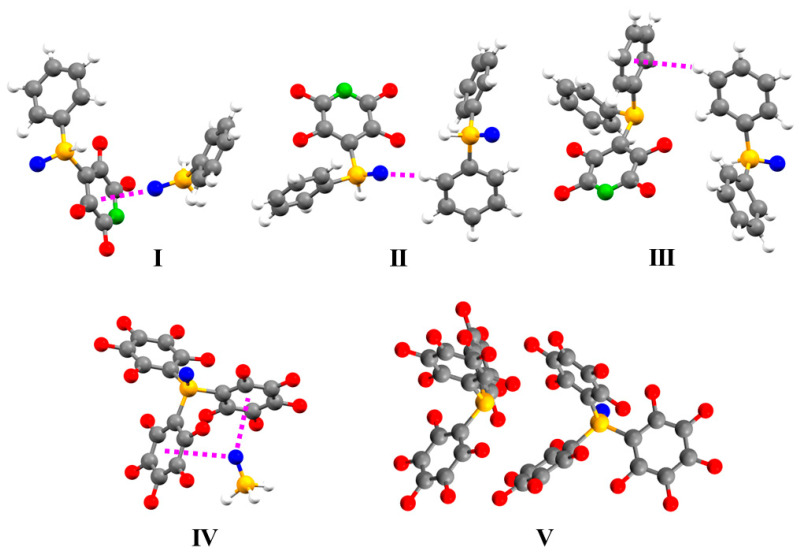
Model systems used to probe the interactions between molecules of 4-(2,3,5,6-tetrafluoropyridyl)diphenylphosphine oxide, **1**, (**I**–**IV**) and tris(pentafluorophenyl)phosphine oxide, **2**, (**V**) within a chain.

To probe the intermolecular interactions, further calculations were performed on model systems selected to include just one intermolecular contact, using the atomic positions of the experimentally determined structures of **1A** and **1B** (Figure 9). The lone pair–π interaction is modelled by **I**, the C–H∙∙∙O interaction by **II**, and the C–H∙∙∙arene interaction by **III**. Whilst the energies of the interactions for the model systems were not expected to be identical to those of the experimentally determined crystal structure, the differences were not expected to be large. At the very least, the relative order of the strengths of the interactions could be determined. It was also not expected that the energies of interactions are purely additive as the compounds are changed, but again, the differences were not expected to be large. The results, summarized in Table 4, indicate this to be the case; the sum of the energies of the individual interactions are within 10% of those of the energies calculated for **1**. The energies of the interactions are similar, with the strengths decreasing in the order lone pair–π > C–H∙∙∙arene > C–H∙∙∙O. Despite the difference in P=O bond lengths and O∙∙∙arene distances, the energies of the lone pair–π interactions are almost identical.

**Figure 10:**
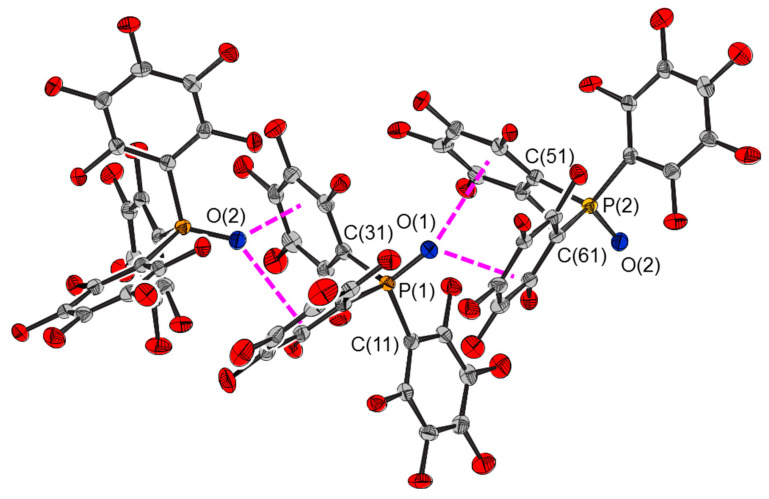
Intermolecular interactions between molecules of tris(pentafluorophenyl)phosphine oxide, **2**, within a chain.

The crystal structure of **2** (Figure 10) possesses short contacts between adjacent molecules of opposite conformation arranged in zig-zagged chains parallel to the *c* axis (the P=O bonds of **2A**, that of P(1), and **2B**, that of P(2), are inclined at 68.3(1) and 31.3(1)° to the *c* axis respectively). The oxygen atom of one molecule is directed towards, and close to, faces of two of the pentafluorophenyl rings of an adjacent non-equivalent molecule, suggesting lone pair–π interactions [22,23,24]. The interaction between pairs of molecules of **2** were calculated to be −99 and −100 kJ mol^−1^. The lone pair–π interactions were modelled by **IV** and interactions between the pentafluoroarene rings by **V**. The energies of the interactions for these models are also given in Table 4. 

Whereas in the structure of **2** the oxygen atoms interact only with pentafluorophenyl rings of a symmetry independent molecule, those in the structure of **1** interact with the tetrafluoropyridyl group of a symmetry independent molecule and the phenyl ring of another molecule. For one molecule of **1**, this is a symmetry-related molecule, but for the other, it is the symmetry independent molecule. This subtle difference appears to account for the large difference in P=O distances for the molecules of **1**, whilst those of **2** are identical. 

## 3. Materials and Methods 

DFT calculations using the long-range corrected functional ωB97XD [19] method with the 6-311G++(2d,2p) basis set were performed using Gaussian 09 [28]. The energies of interaction were calculated as the difference between the energy of the species and the sum of those of its components. The C–H bonds of the experimental structures were normalized to 1.083 Å [29], before single point energy calculations were performed. In the model compounds, the P–H bonds are 1.350 Å [29]. Harmonic vibrational frequencies were calculated to confirm that the optimized structures were minima.

Non-covalent interactions calculations were undertaken with a locally developed software program, Bonder, in combination with the VMD package [30] for visualization. Bonder gives equivalent results to NCIplot [31], but with reduced computational cost. A detailed explanation of NCI is available [32]. 

## Figures and Tables

**Table 1:** Selected bond and intramolecular distances (Å) and angles (°) for 4-(2,3,5,6-Table 1.

Distance or Angle	Molecule A	Molecule B	Calculated
P=O	1.441(3)	1.466(2)	1.478
P–C(11)	1.792(4)	1.788(3)	1.805
P–C(21)	1.798(4)	1.798(3)	1.807
P–C(31)	1.832(3)	1.836(4)	1.851
O=P–C(11)	112.2(2)	112.7(1)	112.9
O=P–C(21)	113.2(2)	114.0(1)	115.2
O=P–C(31)	113.0(2)	110.3(1)	110.8
O=P–C(31)–C(32)	−130.1(3)	128.3(3)	−112.8
O=P–C(31)–C(36)	45.9(3)	−48.9(3)	63.8
O=P–C(11) –C(12)	−128.3(1)	−26.9(3)	22.2
O=P–C(11) –C(16)	51.0(3)	149.6(3)	−156.6
O∙∙∙C(16A/12B)	3.138(5)	3.063(4)	3.053
O∙∙∙H			2.614
O∙∙∙H–C(16A/12B)			103.5
O=P–C(21)–C(22)	31.8(3)	−44.1(3)	24.6
O=P–C(21)–C(26)	−147.4(3)	136.2(3)	−156.2
O∙∙∙C(22)	3.109(5)	3.181(5)	3.121
O∙∙∙H			2.693
O∙∙∙H–C(22)			103.2
C(11A)∙∙∙C(26A) / C(16B)∙∙∙C(21B)	3.168(4)	3.131(6)	3.219
C–H∙∙∙C_6_H_5_^plane a^			2.283

^a^ C_6_H_5_^plane^ is the plane defined by the six carbon atoms of the phenyl ring.

**Table 2:** Selected bond and intramolecular distances (Å) and angles (°) for Table 2.

Distance or Angle	Molecule A	Molecule B	Calculated
P=O	1.467(2)	1.467(2)	1.466
P–C	1.812(2)	1.811(2)	1.822
	1.821(3)	1.813(2)	1.822
	1.826(2)	1.821(2)	1.833
O=P–C	111.5(1)	110.4(1)	111.2
	112.5(1)	114.0(1)	113.1
	115.1(1)	114.3(1)	115.0
O=P–C–C	−1.3(2), 177.4(2)	2.5(2), −173.6(2)	3.3, −173.0
	−50.0(2), 131.8(2)	48.4(2), −136.9(2)	48.8, −130.9
	−56.6(2), 116.4(2)	57.8(2), −114.6(2)	61.9, −110.1
O∙∙∙F	2.800(2)	2.758(2)	2.787

**Table 3:** Selected intermolecular distances (Å) and angles (°) for 4-(2,3,5,6-tetrafluoropyridyl)diphenylphosphine oxide, **1.**

**Within Chains**			
O(1A)···C_5_F_4_N^plane 1^	2.787(5)	O(1B)···C_5_F_4_N^plane^	2.920(5)
O(1A)···C_5_F_4_N^cent. 2^	2.818(5)	O(1B)···C_5_F_4_N^cent.^	2.945(5)
P=O(1A)···C_5_F_4_N^cent.^	141.3(2)	P=O(1B)···C_5_F_4_N^cent.^	136.9(1)
O(1A)···C(26B)	3.363(5)	O(1B)···C(12A)	3.331(4)
P=O(1A)···C(26B)	120.0(2)	P=O(1B)···C(12A)	120.7(1)
O(1A)···C(26B)–C(21B)	128.6(2)	O(1B)···C(12A)–C(11A)	129.2(2)
O(1A)···C(26B)–C(25B)	109.7(2)	O(1B)···C(12A)–C(13A)	111.2(2)
C(13A)···C_6_H_5_^plane^	3.685(6)	C(25B)···C_6_H_5_^plane^	3.810(6)
C(13A)···C_6_H_5_^cent.^	4.220(6)	C(25B)···C_6_H_5_^cent.^	4.068(6)
C(12A)–C(13A)···C_6_H_5_^cent.^	104.3(2)	C(24B)–C(25B)···C_6_H_5_^cent.^	133.6(3)
C(14A)–C(13A)···C_6_H_5_^cent.^	131.2(3)	C(26B)–C(25B)···C_6_H_5_^cent.^	105.7(2)
**Between Chains**			
O(1A)···C(15A)	3.661(4)	O(1B)···C(23A)	3.432(5)
P=O(1A)···C(15A)	125.2(2)	P=O(1B)···C(23A)	122.9(1)
O(1A)···C(15A)–C(14A)	124.5(3)	O(1B)···C(23A)–C(22A)	119.5(3)
O(1A)···C(15A)–C(16A)	113.9(2)	O(1B)···C(23A)–C(24A)	119.3(3)

^1^ C_6_H_5_^plane^ is the plane defined by the six carbon atoms of the phenyl ring. ^2^ C_6_H_5_^cent.^ is the centroid of the six carbon atoms of the phenyl ring.

**Table 4:** Calculated energies of interaction between molecules of **1** and **2**, and model systems.

Compound or Model Structure	Energy kJ mol^−1^	
	A → B	B → A
**1**	−70	−71
**I**	−28	−29
**II**	−23	−21
**III**	−24	−23
		
**2** ^**1**^	−99	−100
**IV** ^**1**^	−38	−38
**V** ^**1**^	−60	−58

^1^ Molecule A is that comprising P(1) and O(1), and molecule B is that comprising P(2) and O(2).
